# The first complete chloroplast genome of *Pteris vittata* (Pteridaceae), an arsenic hyperaccumulating fern

**DOI:** 10.1080/23802359.2018.1501316

**Published:** 2018-08-17

**Authors:** Hairuo Zeng, Mingyu Li, Ruixiang Xu, Shanshan Liu, Zhen Wang, Ting Wang, Yingjuan Su

**Affiliations:** a School of Life Sciences, Sun Yat-sen University, Guangzhou, China;; b College of Life Sciences, Nanjing Agricultural University, Nanjing, China;; c College of Life Sciences, South China Agricultural University, Guangzhou, China;; d Research Institute of Sun Yat-sen University in Shenzhen, Shenzhen, China

**Keywords:** Arsenic hyperaccumulating fern, chloroplast genome, phylogenetic analysis, *Pteris vittata*

## Abstract

It is the first report on complete chloroplast genome of *Pteris vittata*, an arsenic hyperaccumulating fern. Its genome size is 154,130 bp, with a typical circular structure including a large single-copy (LSC) (82,623 bp) and a small single-copy (SSC) (20,957 bp) regions separated by a pair of inverted repeats (25,275 bp each). The plastome encodes 132 genes, including 87 protein-coding genes, 35 tRNA genes, eight rRNA genes, and two pseudogenes. The overall Guanine+Cytosine (GC) content is 41.7% and GC content in the IR regions is higher than in the LSC and SSC regions. Maximum likelihood (ML) tree indicated that *P. vittata* was clustered with *Ceratopteris richardii*.

*Pteris vittata*, commonly known as Chinese brake or ladder fern, is a terrestrial fern belonging to Pteridaceae. It has erect rhizomes and dark straw-coloured fronds. As a perennial evergreen species native to China, it is widely distributed in tropics and subtropics of the Old World (Zhang et al. 2013). The fern grows in calcareous soils, on limestone, also on concrete structures and cracks at an altitude of below 2000 m (Zhang et al. 2013). It is not only a traditional Chinese medicine used for influenza and dysentery (Xie 1996) but also a famous hyperaccumulator of arsenic in phytoremediation (Cesaro et al. 2015). In addition, relationships among some groups in Pteridaceae are subjected to be further classified (Christenhusz and Chase 2014; Ruhfel et al. 2018). Therefore, acquirement of the complete chloroplast of *P. vittata* has important implication for investigating role of chloroplast in arsenic accumulation and deeper phylogenetic relationships.

We isolated total genomic DNA from the fresh leaves of *P. vittata* collected from South China Botanical Garden, Chinese Academy of Sciences (23°11′3.56″N, 113°21′43.28″E), using Tiangen Plant Genomic DNA Kit (Tiangen Biotech Co., Beijing, China) according to the instructions of the manufacturer. Voucher specimen was deposited in the Herbarium of Sun Yat-sen University (SYS; voucher: *SS Liu 201616*). After an average of 300 bp, genomic DNA library was constructed, pair-end sequencing was generated on an Illumina Hiseq 2500 platform. We obtained 7,077,109 raw reads. After trimming the sequences, 5,494,147 clean reads were *de novo* assembled into complete chloroplast genome by Velvet v1.2.07 (Zerbino and Birney 2008). The protein-coding genes, tRNA genes, and rRNA genes were predicted using DOGMA (Wyman et al. 2004) and tRNAscan-SE (Schattner et al. 2005). Maximum likelihood (ML) analysis was performed through RAxML v8.0 (Stamatakis 2014) with 1000 bootstrap replicates using 11 ferns including *Dipteris conjugate* as an outgroup aligned with MAFFT v.7.221 (Katoh and Standley 2013).

The whole chloroplast genome of *P. vittata* (GenBank Accession Number: MH500228) has a total length of 154,130 bp, with 41.7% GC content. It is a typical quadripartite structure with a large single-copy (LSC) region of 82,623 bp, a small single-copy (SSC) region of 20,957 bp, and a pair of inverted repeats (IRa and IRb) of 25,275 bp. The plastome encodes 132 genes, including 87 protein-coding genes, 35 tRNA genes, eight rRNA genes, and two pseudogenes (*ndhB* and *cemA*). The IR regions have higher GC content (45.0%) than the LSC (40.6%) and SSC (37.7%) regions. Most genes occur as a single copy, except for four protein-coding genes, six tRNA genes, and four rRNA genes, which are duplicated in the IR regions. In addition, only three genes (*ycf3*, *clpP*, and *rps12*) have two introns. ML tree indicated that *P. vittata* formed a close relationship with *Ceratopteris richardii* with 100% bootstrap support *values* (Figure 1). The complete chloroplast (cp) genome of *P. vittata* will provide valuable molecular data for further phylogenetic studies, as well as fundamental information to survey mechanism of arsenic hyperaccumulation.

**Figure 1.**
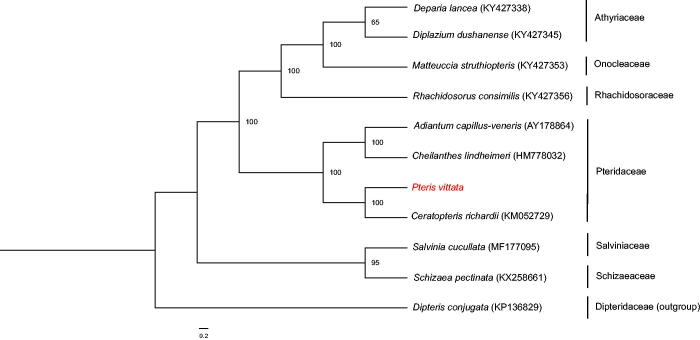
Maximum likelihood tree based on complete chloroplast genome sequences of *Pteris vittata* and other 10 fern including *Dipteris conjugate* as an outgroup. The numbers on the node are referred as bootstrap values based on 1000 bootstrap replicates.
